# Fezf2 promotes neuronal differentiation through localised activation of Wnt/β-catenin signalling during forebrain development

**DOI:** 10.1242/dev.115691

**Published:** 2014-12-15

**Authors:** Siwei Zhang, Jingjing Li, Robert Lea, Kris Vleminckx, Enrique Amaya

**Affiliations:** 1 The Healing Foundation Centre, Faculty of Life Sciences, University of Manchester, Manchester M13 9PT, UK; 2 Department for Biomedical Molecular Biology, Ghent University, B-9052 Ghent, Belgium

**Keywords:** Fezf2, Wnt signalling, *Xenopus*, Forebrain development

## Abstract

Brain regionalisation, neuronal subtype diversification and circuit connectivity are crucial events in the establishment of higher cognitive functions. Here we report the requirement for the transcriptional repressor Fezf2 for proper differentiation of neural progenitor cells during the development of the *Xenopus* forebrain. Depletion of Fezf2 induces apoptosis in postmitotic neural progenitors, with concomitant reduction in forebrain size and neuronal differentiation. Mechanistically, we found that Fezf2 stimulates neuronal differentiation by promoting Wnt/β-catenin signalling in the developing forebrain. In addition, we show that Fezf2 promotes activation of Wnt/β-catenin signalling by repressing the expression of two negative regulators of Wnt signalling, namely *lhx2* and *lhx9*. Our findings suggest that Fezf2 plays an essential role in controlling when and where neuronal differentiation occurs within the developing forebrain and that it does so by promoting local Wnt/β-catenin signalling via a double-repressor model.

## INTRODUCTION

The vertebrate forebrain, which carries out higher neural functions, is a highly organised and complex structure derived from the anteriormost region of the neural plate. Although the extent of elaboration and the size of the various subdomains of the anterior central nervous system vary between species, the molecular mechanisms that generate the brain, including the patterning of the different forebrain subdomains and subsequent neuronal differentiation within each compartment, are highly conserved amongst vertebrates. Therefore, studies on the early development of the forebrain in zebrafish, frog, chick and mouse embryos have shed light on the conserved developmental programmes that contribute to the formation and development of the vertebrate brain (Wilson and Houart, 2004).

Forebrain development comprises three distinct stages. The first stage is neural induction during gastrulation, which defines both the position and identity of the anterior neuroectoderm. This stage is quickly followed by a second, patterning stage, whereby the anterior neuroectoderm is regionalised into the various forebrain subdomains by means of both transcriptional regulation and signal transduction. The third and final phase of forebrain development, which lasts until adulthood, is associated with regionalised growth of the various forebrain subdomains and concomitant specification, migration and differentiation of the various neuronal subtypes that make up the adult brain (Eagleson et al., 1998; Wilson and Houart, 2004).

Wnt/β-catenin signalling has been reported to play an essential role during the patterning and differentiation stages of the forebrain. During the patterning stage, low or absent Wnt/β-catenin signalling in the anterior region of the forebrain is required for telencephalic specification, whereas high Wnt/β-catenin signalling, together with BMP, FGF and Shh signalling, is important for diencephalic specification (Wilson and Houart, 2004). Later, during the growth and differentiation stage, Wnt/β-catenin signalling is activated in the anterior region, promoting the differentiation of neural stem/progenitor cells (Kondo et al., 2011; Machon et al., 2007; Marinaro et al., 2012; Peukert et al., 2011). Thus, Wnt/β-catenin signalling is highly dynamic, both temporally and spatially, during forebrain development and understanding how this dynamic nature is exquisitely regulated is essential for understanding how the brain is moulded during development. Although it is clear that the establishment of a low-to-high Wnt gradient across the anterior-posterior axis patterns different domains of the forebrain, only a few regulators have been identified that control Wnt/β-catenin activity in the anterior region at the onset of the third stage (Juraver-Geslin et al., 2011; Peukert et al., 2011). Specifically, the mechanisms that lead to Wnt/β-catenin activation during the later phase of forebrain development are currently unknown.

*Fezf2*, which is also known as *fezl/Earmuff*, *too few*, *ZNF312* and *Zfp312*, is a highly conserved gene that encodes a zinc finger transcriptional repressor, which is expressed in the forebrain (Shimizu and Hibi, 2009). *Fezf2* homologues have been identified and studied in *Drosophila* (Weng et al., 2010), zebrafish (Berberoglu et al., 2009; Hashimoto et al., 2000; Levkowitz et al., 2003), mouse (Shimizu and Hibi, 2009; Shimizu et al., 2010) and human (Zhu et al., 2010). All *Fezf2* orthologues encode transcription factors characterised by six DNA-binding C2H2-type zinc fingers and an Engrailed homology 1 (Eh1) repressor motif that interacts with Transducin-like enhancer of split (TLE)-type transcriptional co-repressors (Shimizu and Hibi, 2009). Studies in mouse have shown that *Fezf2*-expressing radial glial cells are multipotent progenitors that generate all major projection neurons and glia of the neocortex (Guo et al., 2013). In addition, *Fezf2* controls neuronal subtype differentiation, including that of subplate neurons (Hirata et al., 2004; Rouaux and Arlotta, 2010), specification of subcortical projection neurons in cortex layer V (Chen et al., 2008) and patterning of the forebrain and olfactory systems (Shimizu and Hibi, 2009). Furthermore, *Fezf2* is required for the establishment of diencephalic subdivisions (Hirata et al., 2006). In *Drosophila*, *Fezf2* restricts the developmental potential of intermediate neural progenitors (Weng et al., 2010). In zebrafish, *fezf2* is co-expressed with neural stem markers in the adult brain (Berberoglu et al., 2009), where it controls the development of monoaminergic neurons (Jeong et al., 2006; Levkowitz et al., 2003) and is involved in patterning of the diencephalon (Jeong et al., 2007). More recently, *Fezf2* has been reported to possess a unique ability to reprogramme postmitotic neurons *in vivo* (De la Rossa et al., 2013; Rouaux and Arlotta, 2013). Notably, although many roles for *Fezf2* have been described, very little is known about the molecular mechanisms underlying its functions during forebrain development.

We have identified *fezf2* as a positive regulator of Wnt/β-catenin signalling in the rostral forebrain, and we have revealed the molecular mechanism by which *fezf2* triggers Wnt signalling and consequent neural progenitor differentiation and forebrain growth in the *Xenopus* embryo. We demonstrate that *fezf2* is expressed in the developing *Xenopus* forebrain. Depletion of *fezf2* in embryos results in arrested neural progenitor differentiation, increased apoptosis, and reduction in forebrain size. We also show that *fezf2* promotes Wnt/β-catenin signalling at the differentiation stage, and that this activity is required for proper development of the forebrain. We further reveal that Fezf2 interacts with co-repressors of the Groucho family and, through its repressor activity, restricts the expression of *lhx2* and *lhx9*, which encode two negative regulators of Wnt/β-catenin signalling in the forebrain, thus explaining its Wnt-promoting role. Taken together, we conclude that *fezf2* initiates proper neuronal differentiation in the forebrain by promoting localised Wnt/β-catenin signalling through a double-repressor model.

## RESULTS

### *fezf2* is expressed in the anterior forebrain during early development

We isolated *fezf2* from an *in vivo* large-scale gain-of-function screen aimed at identifying novel regulators of several signal transduction pathways during early *Xenopus* development (Zhang et al., 2013)*.* Subsequent qPCR analyses revealed that *fezf2* expression begins at the early gastrula stage (stage 10.5), reaching a maximum at the mid-neurula stage (stage 15), at which time its expression decreased slightly and plateaued thereafter (supplementary material Fig. S1). This pattern was very similar to that obtained from whole-exome deep sequencing (Tan et al., 2012). We then assessed the spatial expression pattern of *fezf2* using whole-mount *in situ* hybridization. These data revealed that *fezf2* is expressed in the prospective anterior neural region and presumptive forebrain region from the early neurula stages (stage 15) (supplementary material Fig. S2). At the tailbud stage (stage 28), *fezf2* was expressed in the telencephalon, ventral diencephalon and the eye vesicle (supplementary material Fig. S2). At early tadpole stages (stage 35), the expression of *fezf2* remained restricted to the forebrain and eye vesicle (supplementary material Fig. S2).

### *fezf2* is required for proper neuronal differentiation within the forebrain

**Fig. 1.**
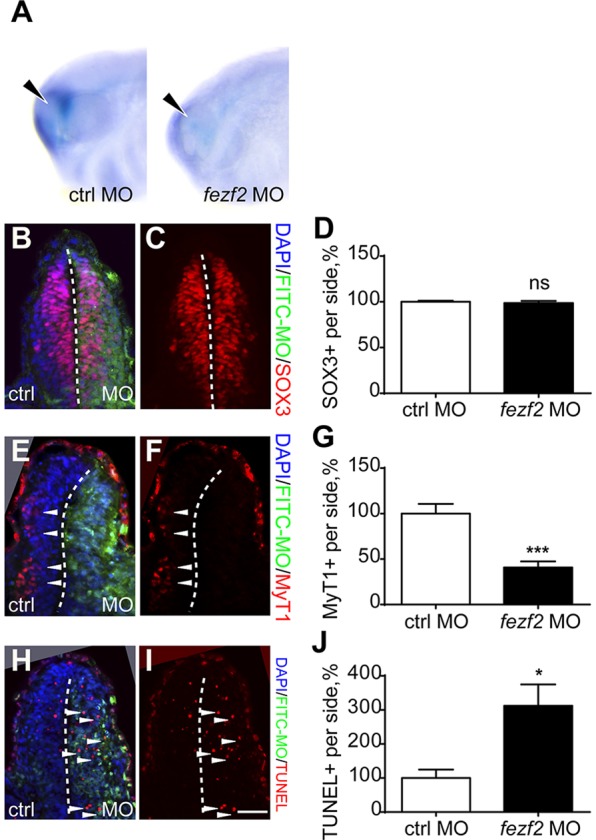
***fezf2* knockdown leads to defects in forebrain neuronal differentiation.** (A) Whole-mount *in situ* hybridisation for *arx* in control MO (20/20) or *fezf2* MO (15/18) injected *Xenopus* embryos. Arrowhead indicates the forebrain. (B-J) One blastomere at the 2-cell stage was injected with *fezf2* MO and embryos were sectioned at stage 30 transversely across the forebrain, and stained for Sox3 (B,C), MyT1 (E,F) or TUNEL (H,I). FITC staining identifies the injected side (B,E,H). Arrowheads indicate MyT1^+^ (E,F) or TUNEL^+^ (H,I) cells. (D,G,J) Statistical analysis of Sox3^+^ (*n*=4 embryos), MyT1^+^ (*n*=6 embryos) and TUNEL^+^ (*n*=4 embryos) cells. All control sides have been normalised to 100%. Error bars represent s.e.m. *P<0.05; ***P<0.001; ns, not significant. Scale bar: 25 µm.

To dissect the function of *fezf2*, we first performed a series of knockdown experiments using an antisense morpholino oligonucleotide (MO) targeting the exon 3-intron 3 splice junction of the pre-mRNA (supplementary material Fig. S3A). The knockdown efficiency of this MO was validated using RT-PCR and qPCR (supplementary material Fig. S3A,B). Embryos injected with control MO exhibited normal forebrain development, whereas *fezf2* MO caused significant disruption in the development of the forebrain, as revealed by diminution in the expression of the rostral forebrain-specific marker *arx* (Fig. 1A) as well as the anterior neural markers *otx2* and *pax6* at stage 30 (supplementary material Fig. S4A) (El-Hodiri et al., 2003). Notably, early forebrain patterning was unaffected in *fezf2* morphants, as stage 15 (early neurula) embryos did not exhibit altered expression of the forebrain markers *arx*, *otx2* and *pax6* (supplementary material Fig. S4B).

To characterise the cell types in the forebrain that were affected by loss of *fezf2*, we injected the *fezf2* MO into one cell of 2-cell stage embryos, and then we assessed the effect of this perturbation on specific cell populations in the injected side of the forebrain versus the control side at stage 30. No significant change was observed in the number of Sox3^+^ neural progenitor cells in the *fezf2* MO-injected side versus non-injected side (Fig. 1B-D) (Wang et al., 2006). However, *fezf2* knockdowns resulted in a 45% reduction in the number of differentiating neurons, as assayed by immunostaining for the primary neuronal differentiation marker Myelin transcription factor 1 (MyT1) (Fig. 1E-G) (Bellefroid et al., 1996). We further confirmed a reduction in differentiated neurons by staining with an acetylated β-tubulin antibody, which labels the axons of differentiated neurons (supplementary material Fig. S4La-c). No reduction in either Sox3^+^ or MyT1^+^ cells was observed in embryos injected with a control MO (supplementary material Fig. S4C-E,F-H). Therefore, *fezf2* is required for neuronal differentiation, but is not essential for the maintenance of neural progenitor cell populations.

One possibility for the reduction in the number of differentiated neurons in the forebrain area is that *fezf2* is required for cell survival during differentiation. To address this, we performed TUNEL assays on control versus knockdown sides of the embryos. These experiments revealed that injection of *fezf2* MO, but not the control MO, caused a 3-fold increase in the number of apoptotic cells in the knockdown side versus the control side of the forebrain (Fig. 1H-J; supplementary material Fig. S4I-K). Taken together, we conclude that *fezf2* controls the transition from neuronal progenitors to differentiated neurons, but is not required for the early forebrain patterning events, nor for the maintenance of neural progenitor cells prior to neuronal differentiation.

### *fezf2* promotes Wnt/β-catenin signalling in early embryos

**Fig. 2.**
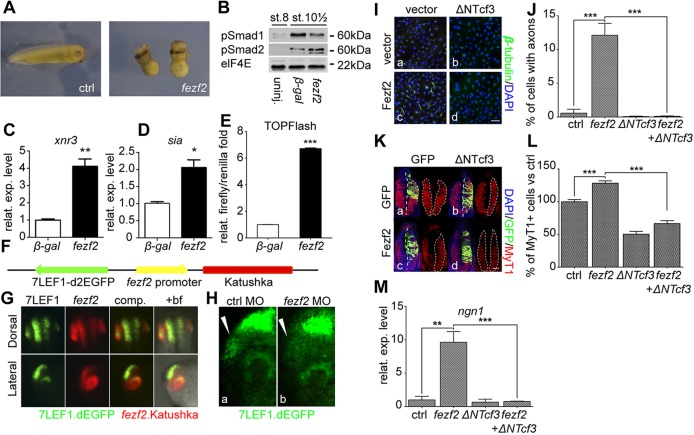
***fezf2* promotes Wnt/β-catenin signalling and induces neuronal differentiation through Wnt/β-catenin *in vitro* and *in vivo*.** (A) *fezf2* misexpression in early *Xenopus* embryos leads to strong dorsoanteriorisation (31/35 embryos examined showed the illustrated phenotype) compared with *lacZ* (β-gal) controls (39/39). (B) *fezf2* misexpression enhances Smad2/3 phosphorylation and inhibits Smad1/5/8 phosphorylation as assessed in western blots. Blastula stage (st. 8) indicates the pre-activation state. Elongation factor 4E (elF4E) was used as a loading control. (C,D) qPCR shows that *fezf2* promotes the expression of *xnr3* (C) and *sia* (D) in early embryos (*n*=3 replicates). (E) TOPFlash assay shows that *fezf2* promotes Wnt/β-catenin signalling (*n*=4 replicates). (F-H) *fezf2* expression colocalises with active Wnt signalling in the forebrain. (F) The transgenic construct. (G) Dorsal and lateral views of stage 30 embryos; GFP signal for Wnt activity (green); Katushka signal for *fezf2* expression (red); +bf, merged image with bright-field. (H) Knockdown of *fezf2* reduces Wnt activity in the forebrain as assessed by expression of the 7LEF-dEGFP F1.1 Wnt reporter line. Arrowhead indicates the diencephalon. (I) The Wnt inhibitor ΔNTcf3 antagonises Fezf2-induced neuronal differentiation in mouse neuronal progenitors, as assessed by the induction of axonogenesis. (J) Statistics of I (*n*=4 replicates). (K,L) Electroporation experiments show that the Wnt inhibitor ΔNTcf3 antagonises Fezf2-induced neuronal differentiation in the tadpole forebrain. (K) Transverse sections of the forebrain area of stage 30 embryos electroporated correspondingly and stained for MyT1 (red), GFP (green) and with DAPI (blue). Left images, merge; right images, MyT1 alone. (L) Statistics of K (*n*=5 embryos). Control side is normalised to 100%. (M) qPCR analysis shows that the Wnt inhibitor ΔNTcf3 antagonises Fezf2-induced *ngn1* expression in stage 20 animal cap explants (*n*=3 replicates). In all qPCR analyses, *ribosomal protein L8* (*rpl8*) was used as an internal control. *P<0.05, **P<0.01, ***P<0.001. Error bars represent s.e.m. Scale bars: 100 µm in I; 50 µm in K.

We next investigated the mechanism(s) by which Fezf2 acts during development. It was first noted during the functional screen (Zhang et al., 2013) that embryos injected with *fezf2* mRNA are significantly dorsoanteriorised, resembling LiCl-treated embryos (Kao and Elinson, 1988; Kao et al., 1986) or those with excessive Wnt/β-catenin signalling (Smith and Harland, 1991) (Fig. 2A). Moreover, injection of *fezf2* mRNA into early *Xenopus* embryos resulted in an increase in Smad2/3 phosphorylation, which is a measure of TGFβ/Nodal signalling, and a decrease in Smad1/5/8 phosphorylation, which is a measure of BMP signalling (Fig. 2B) (Zhang et al., 2013), changes that are similar to those seen after injection of *wnt8* mRNA in early embryos (supplementary material Fig. S5A). Together, these phenotypic and signalling changes suggested that *fezf2* overexpression might lead to hyperactivation of Wnt/β-catenin signalling.

To confirm whether *fezf2* is able to activate Wnt/β-catenin signalling, we examined if injection of *fezf2* mRNA is able to induce the expression of the immediate Wnt-responsive genes *xnr3* and *siamois* (*sia*) (Sheldahl et al., 1999). Indeed, overexpressing *fezf2* led to a robust increase in the expression level of these two Wnt-responsive genes in early embryos (Fig. 2C,D). In addition, early gastrula stage embryos overexpressing *fezf2* exhibited quantitatively higher levels of expression of *goosecoid* (*gsc*) and *chordin* (*chd*), two additional Wnt/β-catenin-responsive genes (Pierce and Kimelman, 1995), and led to an expansion of the expression domains of these two genes beyond the dorsal organizer region (supplementary material Fig. S5B-G). By contrast, expression of the ventral markers *vent1* (Sander et al., 2007) and *bmp4* (Baker et al., 1999) was downregulated in *fezf2-*overexpressing embryos, further confirming that misexpression of *fezf2* leads to a strong dorsoanteriorisation of embryos (supplementary material Fig. S5H-M).

The translocation and nuclear accumulation of β-catenin is a direct indicator of Wnt/β-catenin signalling activation (Cadigan and Nusse, 1997). Hence, we examined nuclear accumulation of β-catenin in control versus *fezf2* mRNA-injected embryos, using a DAPI mask to specifically reveal the presence of nuclear β-catenin (Schohl and Fagotto, 2002). In control embryos, nuclear β-catenin was preferentially enriched in the dorsal blastoporal lip of gastrula stage embryos (supplementary material Fig. S5Na,a′), consistent with previous findings (Schohl and Fagotto, 2002). However, in *fezf2* mRNA-injected embryos, a much stronger nuclear accumulation of β-catenin was found throughout the embryo, suggesting widespread hyperactivity of Wnt/β-catenin signalling (supplementary material Fig. S5Nb,b′). In addition, injection of *fezf2* mRNA into one of the two ventral blastomeres at the 4-cell stage induced axis duplication with complete head in more than 75% of embryos (supplementary material Table S1 experiment I, Fig. S5O,P), as is often observed following ectopic activation of Wnt/β-catenin signalling (Sokol et al., 1991). Furthermore, this *fezf2*-induced secondary axis induction could be antagonised by co-injecting an N-terminally truncated dominant-negative form of Tcf3 (ΔN51-Tcf3) (supplementary material Table S1 experiments II-1 to II-4) or *nlk1* mRNA, a direct inhibitor of Wnt/β-catenin signalling (supplementary material Table S1 experiments II-5 and II-6) (Ishitani et al., 1999; Molenaar et al., 1996). Thus, *fezf2* misexpression leads to robust hyperactivation of Wnt/β-catenin signalling.

In order to observe a more direct effect of *fezf2* on Wnt/β-catenin signalling, we performed *in vivo* luciferase assays using a Wnt-responsive construct, TOPFlash (Veeman et al., 2003). Co-injection of *fezf2* mRNA with the TOPFlash DNA construct caused an 8-fold increase in luciferase activity over the *lacZ* (β-gal) control (Fig. 2E), whereas *fezf2* mRNA together with the FOPFlash construct, which contains mutated TCF consensus binding motifs, failed to exhibit an increase luciferase activity (supplementary material Fig. S5Q). These experiments confirmed that *fezf2* overexpression activates Wnt/β-catenin signalling in early embryos.

A previous investigation has suggested that *Fezf2* negatively regulates Wnt/β-catenin signalling in mouse embryonic stem cells (mESCs) by repressing the expression of Wnt ligands (Wang et al., 2011). We tested the expression of several canonical Wnt signalling-related ligands in control versus *fezf2*-expressing animal cap explants. To induce anterior neuroectoderm, we injected *chd* mRNA, which encodes a potent BMP antagonist, into early embryos and allowed the explants to develop until stage 15 (Sasai et al., 1995). The expression of *wnt1* was slightly increased in *fezf2*-expressing animal cap explants, whereas the expression of *wnt3a* and *wnt8b* remained unchanged (supplementary material Fig. S5R).

### Expression of *fezf2* colocalises with and is functionally required for active Wnt/β-catenin signalling in the forebrain

We next asked whether *fezf2* expression in the forebrain correlates with active Wnt/β-catenin signalling. We isolated ∼3 kb of the *fezf2* proximal promoter region and used it to drive the expression of Katushka in transgenic embryos (Shcherbo et al., 2007). In addition, we co-integrated a Wnt reporter cassette, 7LEF-dEGFP, with the *fezf2*-Katushka cassette using our recently developed pTransgenesis system to generate the transgenic embryos (Love et al., 2011; Tran et al., 2010), which allowed us to observe the state of activation of Wnt signalling (Denayer et al., 2006) and *fezf2* promoter activity in the same embryos (Fig. 2F). The resulting transgenic embryos exhibited strong colocalisation of dEGFP signal (Wnt) and Katushka signal (*fezf2*) in the telencephalic and diencephalic areas, although a broader *fezf2* expression was observed in the eye, which might reflect the much longer half-life of Katushka relative to dEGFP (Fig. 2G). In addition, injection of *fezf2* MO into 7LEF-dEGFP F1.1 transgenic embryos led to a significant decrease in dEGFP expression (i.e. in active Wnt signalling) at stage 32 in the forebrain (Fig. 2Ha, arrowhead), compared with control MO-injected embryos (Fig. 2Hb, arrowhead) (Tran et al., 2010). These data indicated that *fezf2* expression not only colocalises with active Wnt/β-catenin signalling, but is also functionally required for maintaining active Wnt signalling in the forebrain.

### *fezf2* overexpression promotes forebrain neuronal differentiation through Wnt/β-catenin signalling

Fezf2 has been reported to induce neuronal differentiation in mESCs, as well as to induce the differentiation of striatal progenitors into telencephalic precursors and corticofugal neurons (Rouaux and Arlotta, 2010; Wang et al., 2011). Based on our findings, we next asked whether *fezf2* induces forebrain neuronal differentiation through its ability to activate Wnt signalling. We began by transfecting a construct carrying the mouse *Fezf2* gene (pCS107-*Fezf2*) into an immortalised mouse C17.2 neural stem cell line, together with either empty vector (pCS2) or the Wnt-inhibitory truncated *ΔTcf3* construct (pCS107-*ΔN51-Tcf3*) (Molenaar et al., 1996; Roose et al., 1998), followed by an assessment of neuronal differentiation in these cells (Mi et al., 2005). Transfection of the *Fezf2* construct alone induced a significant proportion of the neural stem cells to differentiate into neurons, as assessed by evaluating the formation of neuronal β-tubulin^+^ axons (Fig. 2Ia,c,J). However, this induction was antagonised by co-transfecting the Wnt-inhibitory *ΔN51-Tcf3* construct, but not by a control empty vector (Fig. 2Ia,d,J). Transfecting the neural stem cells with the *ΔN51-Tcf3* construct alone also had no effect (Fig. 2Ib,J). These results indicated that *fezf2* induces neuronal differentiation *in vitro*, and that this induction requires Wnt/β-catenin signalling.

To examine whether Fezf2 induces neuronal differentiation through Wnt/β-catenin signalling *in vivo*, we electroporated a construct containing CMV promoter-driven *fezf2* (pCS107 backbone) with or without the Wnt-inhibitory construct (*ΔN51-Tcf3*) into the third ventricle of stage 26 *X. laevis* embryos, and allowed them to develop until stage 31 for analysis. Electroporation of pCS107-*fezf2* significantly increased the number of differentiated primary neurons (MyT1^+^) in the forebrain area, as found in previous studies (Fig. 2Kc,L) (Rouaux and Arlotta, 2010; Wang et al., 2011). However, co-electroporation of pCS107-*fezf2* with pCS107-*ΔN51-tcf3* failed to increase the number of MyT1^+^ cells, suggesting that Fezf2 requires Wnt/β-catenin signalling to induce neuronal differentiation *in vivo* (Fig. 2Ka,d,M).

Previous studies have shown that *neurogenin 1* (*ngn1*), a gene involved in neuronal differentiation, is inducible by Wnt/β-catenin signalling and is *fezf2* responsive (Hirabayashi et al., 2004; Jeong et al., 2006). Therefore, we tested whether the *fezf2*-induced activation of *ngn1* expression is dependent on Wnt/β-catenin signalling. *fezf2* mRNA was injected into *Xenopus* embryos at the 1- to 2-cell stage with or without *ΔN51-tcf3* mRNA. Animal cap explants were dissected at stage 8 and collected at stage 20 to assess the expression of *ngn1* by qPCR (supplementary material Fig. S5S). Misexpression of *fezf2* mRNA induced *ngn1* expression; however, this induction was attenuated when *fezf2* mRNA was co-injected with *ΔN51-tcf3* mRNA. Injection of either *lacZ* mRNA (control) or *ΔN51-tcf3* mRNA alone had no effect on *ngn1* expression in the animal cap explants (Fig. 2M). These results confirmed that Fezf2 promotes neuronal differentiation *in vivo* in a Wnt-dependent manner.

### *fezf2* functions as a transcriptional repressor and governs forebrain neurogenesis through its ability to activate Wnt signalling

**Fig. 3.**
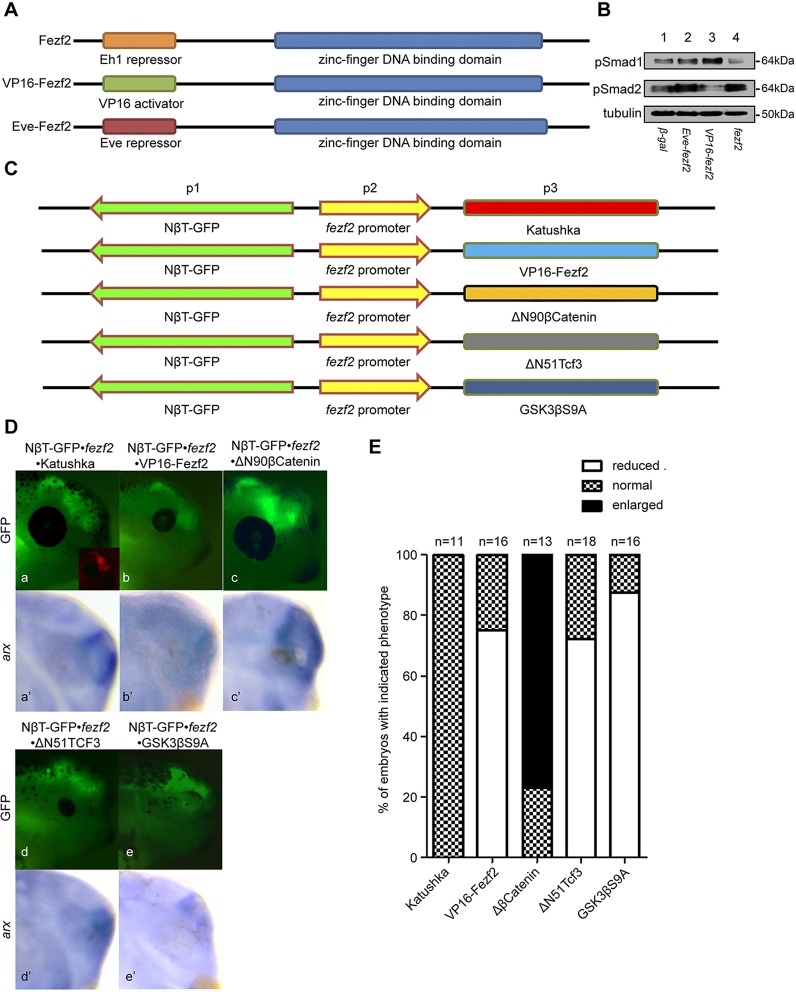
**The Fezf2-regulated endogenous level of Wnt/β-catenin signalling governs forebrain neurogenesis.** (A) Different Fezf2 constructs. Different N-terminal domains (Eh1-repressor, VP16 activator or Eve repressor) are shown in different colours. The zinc-finger DNA-binding domain is shown in blue. (B) Western blot of gastrula stage *Xenopus* embryos injected with nuclear *lacZ* (control), *eve*-*fezf2*, *VP16*-*fezf2* and *wt-fezf2* and assayed for phosphorylated Smad1 or Smad2 and α-Tubulin (loading control). (C) pTransgenesis system transgenic constructs to assess the impact of Fezf2 and/or Wnt activities on forebrain development. (D) Expression of NβT-GFP (a-e, stage 40 embryo) and *arx* (a′-e′, stage 30 embryo) in the forebrain of transgenic embryos harbouring the transgenes shown in C. Inset (a) shows the fluorescence from Katushka (red). (E) Quantification of neural tissue growth phenotypes from D.

We next asked whether endogenously expressed *fezf2* is involved in activating neuronal differentiation through its capacity to activate Wnt signalling. Fezf2 contains two functional domains: a DNA-binding zinc finger domain, and an Eh1 repressor domain that interacts with TLEs (Buscarlet and Stifani, 2007). We therefore constructed an antimorphic form of Fezf2 (VP16-Fezf2) by replacing its Eh1 domain with the transcriptional activator domain of the viral protein VP16, generating a fusion protein that would be expected to function as a transcriptional activator (de Souza et al., 1999; Ferreiro et al., 1998; Latinkic and Smith, 1999; Onichtchouk et al., 1998). We also replaced the Eh1 domain with the transcriptional repressor domain of *Drosophila* Even-skipped (Eve) (Han and Manley, 1993), and this construct (Eve-Fezf2) would be expected to repress transcription of its target genes, similar to wild-type Fezf2 (Fig. 3A). Injection of mRNA encoding Eve-Fezf2 increased Smad2/3 phosphorylation, similar to that of wild-type Fezf2 (Fig. 3B, lanes 2 and 4), although it failed to inhibit the phosphorylation of Smad1, which might be attributed to the slight differences between the two repressor domains. By contrast, VP16-Fezf2 led to strong ventralisation of embryos (supplementary material Fig. S6A-C), together with a reversed pattern of Smad1/5/8 and Smad2/3 phosphorylation (Fig. 3B, lanes 3 and 4). Hence, we validated the functionality of the antimorphic Fezf2 construct and confirmed that Fezf2 acts as a transcriptional repressor in *Xenopus* embryos.

We next investigated whether endogenously augmented Wnt/β-catenin signalling within the *fezf2*-expressing regions affects forebrain development. We inserted a cassette comprising a neural-specific β-tubulin promoter driving tauGFP (NβT-tauGFP) into the p1 site of the pTransgenesis system to assess differentiated neural tissue in transgenic embryos (Love et al., 2011). The 3.5 kb *fezf2* promoter was placed in the p2 site in the opposite orientation to the p1 NβT-tauGFP cassette to minimise potential promoter interference. The p3 cassette was placed directly downstream of the *fezf2* promoter so that any gene within the cassette would be expressed under the control of this promoter (Fig. 3C) (Donnelly et al., 2001). In addition to NβT-tauGFP, the forebrain-specific marker *arx* was also used to monitor the affected neural tissue in different transgenic embryos. The control transgenic construct with Katushka placed in the p3 position resulted in normal forebrain development (Fig. 3Da,a′,E; supplementary material Fig. S6Da). However, the antimorphic VP16-Fezf2 transgenic embryos displayed a significant reduction in *arx* staining, NβT-tauGFP marked neural tissue, and decreased eye size (Fig. 3Db,b′,E; supplementary material Fig. S6Db), similar to transgenic embryos expressing the Wnt-antagonising ΔN51-Tcf3 and GSK3βS9A constructs, which had reduced Wnt activity in *fezf2*-expressing regions (Fig. 3Dd-e′,E; supplementary material Fig. S6Dd,e). By contrast, transgenic embryos expressing the Wnt-agonising ΔN90-β-catenin construct demonstrated expansion of *arx* staining, excessive growth of differentiated neural tissue, and enlarged eyes, suggesting that elevated Wnt activity promotes the growth of neural tissue within the forebrain (Juraver-Geslin et al., 2011) (Fig. 3Dc,c′,E; supplementary material Fig. S6Dc). These results confirmed that the antimorphic Fezf2 acts as a negative regulator of Wnt signalling, and that proper Wnt signalling in *fezf2*-expressing areas is crucial for normal forebrain development *in vivo*.

### Fezf2 physically interacts with Groucho family co-repressors via its N-terminal Eh1 domain

**Fig. 4.**
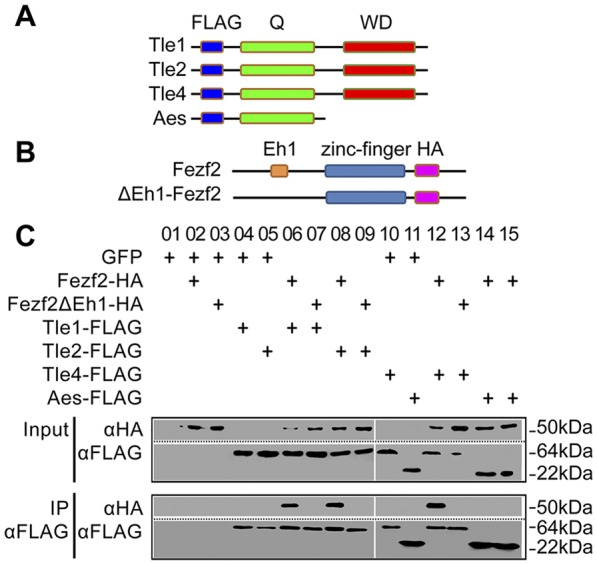
**Fezf2 functions through interaction with members of Groucho family.** (A) Tle1, Tle2, Tle4 and Aes constructs. Note that Aes lacks the protein-interaction WD domain. (B) Wild-type Fezf2 and ΔEh1-Fezf2 with a mutated Eh1 domain. (C) Immunoprecipitation of extracts from *Xenopus* embryos injected with different combinations of the indicated mRNAs, showing that Fezf2 interacts with Tle1, Tle2 and Tle4 (lanes 6, 8 and 12) but not Aes (lanes 14, 15). The Eh1 domain is required for the proper interaction between Fezf2 and Tle1/2/4 (lanes 6 and 7, 8 and 9, 12 and 13).

To investigate the mechanism by which Fezf2 promotes Wnt/β-catenin signalling while acting as a transcriptional repressor, we first examined whether Fezf2 can physically interact with TLEs via its Eh1 domain (Buscarlet et al., 2008; Gasperowicz and Otto, 2005). Amongst the four TLEs found in *Xenopus*, three (Tle1, Tle2 and Tle4) possess the Eh1-interacting WD domain (Fig. 4A) (Roth et al., 2010). In an *in vivo* co-immunoprecipitation assay performed with gastrula stage (10.5) embryos, Fezf2 interacted with all three TLEs that possess the Eh1-interacting WD domain (Fig. 4C, lanes 6, 8 and 12). Aes, the only TLE that does not possess a WD domain, did not interact with Fezf2 (Fig. 4C, lane 14). In addition, a mutated Fezf2 with five conserved hydrophobic amino acid residues removed within the Eh1 domain (ΔEh1-Fezf2, Fig. 4B) lost its ability to interact with the TLEs (Fig. 4C, lanes 7, 9 and 13), confirming Eh1 itself as the interaction domain between Fezf2 and TLEs.

Finally, since Tle4 can complex with Tcf and thus is an important component of Wnt signalling, we examined whether Fezf2 affects Wnt signalling by titrating Tle4 away from the Tle4-Tcf complex. We generated an additional *fezf2* mutant (C284S) that has a point mutation in the DNA-binding zinc finger domain but an intact Eh1 domain (Levkowitz et al., 2003). In contrast to wild-type *fezf2*, ventral blastomere injection of *fezf2* C284S mRNA was unable to induce anterior structures or secondary axes (supplementary material Table S1 experiment IV-3) (Levkowitz et al., 2003). Thus, our data provide compelling evidence that Fezf2 interacts with Groucho family co-repressors through its Eh1 domain and acts as a transcriptional repressor.

### Fezf2 represses *lhx2* and *lhx9* expression to promote Wnt/β-catenin signalling in the forebrain area

**Fig. 5.**
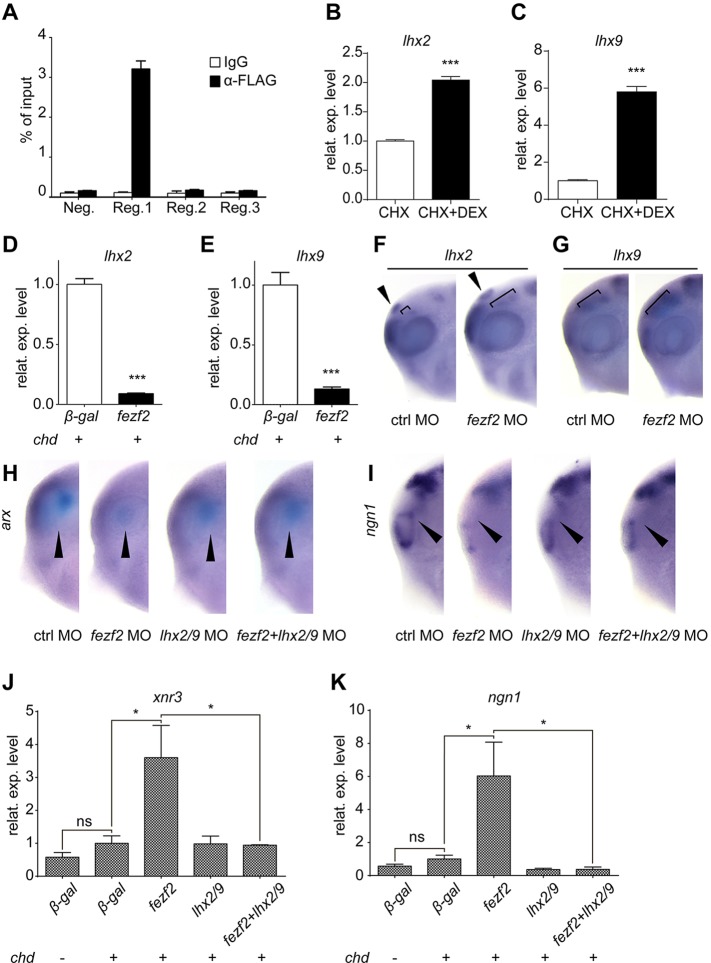
**Fezf2 represses the activity of *lhx2* and *lhx9* in the forebrain*.*** (A) ChIP-qPCR analysis of Fezf2 binding to the *lhx2* promoter. Region 1 showed very high enrichment (*n*=3 replicates). (B,C) qPCR analysis of *lhx2* and *lhx9* expression in p3hGR-VP16-Fezf2-injected animal cap explants aged to stage 12 and treated with CHX alone or CHX+DEX (*n*=3 replicates). (D,E) qPCR analysis of *lhx2* and *lhx9* expression in neuralised animal cap explants aged to stage 20 (*n*=3 replicates). (F,G) *In situ* hybridisation analysis shows that mild knockdown of *fezf2* leads to expansion of the *lhx2* (F) and *lhx9* (G) expression area. Arrowhead indicates the epithalamus; bracket indicates the ventral diencephalon. (H,I) *In situ* hybridisation analysis of *arx* (H) or *ngn1* (I) in stage 28 morphants (lateral views). Arrowheads indicate *arx* or *ngn1* expression. (J) qPCR analysis shows that *lhx2* and *lhx9* antagonise expression of the *fezf2*-induced Wnt-responsive gene *xnr3* in stage 14 animal cap explants (*n*=3 replicates). (K) qPCR analysis shows *lhx2* and *lhx9* antagonise *fezf2*-induced *ngn1* expression in stage 20 animal cap explants (*n*=3 replicates). In all qPCR analyses, *rpl8* was used as internal control. Error bars represent s.e.m. *P<0.05, ***P<0.001; ns, not significant.

To investigate the regulatory mechanism by which *fezf2* activates Wnt/β-catenin signalling within the forebrain, we noted previous reports suggesting that Fezf2 binds to the promoter region of *lhx2* (Chen et al., 2011; Lodato et al., 2014). Furthermore, we noted that *lhx2* and *lhx9* inhibit Wnt/β-catenin signalling in the forebrain (Chen et al., 2011; Peukert et al., 2011). We therefore examined whether Fezf2 promotes Wnt signalling by repressing the expression of *lhx2* and *lhx9*, thus acting in a double-repression model. We first performed ChIP-qPCR experiments in stage 15 embryos to confirm whether Fezf2 directly binds to the promoter region of *lhx2* in *Xenopus*. Since no Fezf2 antibodies were available in *Xenopus*, we utilised a FLAG-tagged version of Fezf2 in *Xenopus* embryos for co-immunoprecipitation with anti-FLAG antibody, a strategy successfully validated by using a FLAG-tagged FoxH1 protein on the *brachyury* promoter (supplementary material Fig. S7A) (Akkers et al., 2012, 2010). We then identified three conserved regions within ∼15 kb upstream of the *lhx2* transcription start site by sequence homology analysis, and then used these regions for ChIP-qPCR analysis (supplementary material Fig. S7B). A high ChIP enrichment was detected around the −12 kb region (Fig. 5A, region 1; supplementary material Fig. S7B), whereas no ChIP enrichments were found in the other two regions tested (Fig. 5A, regions 2 and 3; supplementary material Fig. S7B).

We were unable to perform ChIP-qPCR within the promoter region of *lhx9* as the available sequence data for this region in the *Xenopus tropicalis* genome is incomplete. Instead, we employed an alternate strategy to determine whether Fezf2 directly influences the transcriptional activity of *lhx9*. Antimorphic VP16-Fezf2, if activated, should be able to trigger the expression of Fezf2 direct target genes, even in the absence of protein synthesis. Hence, we made a VP16-Fezf2 construct fused to the 3′-end of human glucocorticoid receptor (hGR) (termed p3hGR-VP16-Fezf2), which can be activated by the addition of dexamethasone (DEX) (Ryan et al., 2004). Reassuringly, we found that animal caps overexpressing p3hGR-VP16-Fezf2 were able to activate the expression of *lhx2* and *lhx9* in the presence of DEX, but not in its absence (supplementary material Fig. S8A,B). Furthermore, we were able to show that the potent protein synthesis inhibitor cycloheximide (CHX) (Saka et al., 2000) had no effect on the expression level of *lhx2* or *lhx9* in animals caps overexpressing p3hGR-VP16-Fezf2 when added alone (supplementary material Fig. S8A,B). Importantly, however, treatment of animals caps overexpressing p3hGR-VP16-Fezf2 with both CHX and DEX led to 2-fold and 6-fold increases in the expression levels of *lhx2* and *lhx9*, respectively (Fig. 5B,C)*.* Thus, p3hGR-VP16-Fezf2 is able to activate the expression of *lhx2* and *lhx9* even in the absence of de novo protein synthesis, providing compelling evidence that both of these genes are direct targets of Fezf2.

We next assessed whether *lhx2* and/or *lhx9* act downstream of *fezf2* during forebrain development. Both *lhx2* and *lhx9* are expressed in the anterior neural ectoderm (supplementary material Fig. S2). Animal cap explants neuralised by *chd* and aged to stage 20 expressed significant levels of both *lhx2* and *lhx9* compared with control *lacZ*-injected embryos, indicating that these explants recapitulate anterior neuroectoderm (supplementary material Fig. S8C,D)*.* However, the expression of both *lhx2* and *lhx9* was inhibited by co-expressing *fezf2* in *chd-*neuralised animal cap explants (Fig. 5D,E), suggesting that Fezf2 is a potent negative regulator of both genes. By contrast, *fezf2* knockdown following injection of 5 ng *fezf2* MO per embryo resulted in an expansion of both the *lhx2* and *lhx9* expression domains in the forebrain area of stage 28 embryos, including an expansion of *lhx2* expression in the epithalamus (Fig. 5F,G; supplementary material Fig. S8E).

We next designed and validated MOs targeting *lhx2* and *lhx9* (supplementary material Fig. S8F-I) and examined whether the activity of *fezf2* could be rescued by simultaneously knocking down both *lhx2* and *lhx9*. We found that, whereas most embryos injected with *fezf2* MO displayed reduced expression of *arx*, embryos injected with *fezf2* and *lhx2*/*lhx9* MOs showed partial rescue in the expression of *arx* at stage 28 (Fig. 5H; supplementary material Fig. S8J). Furthermore, no significant changes in *arx* expression were observed in embryos injected with *lhx2*/*lhx9* MOs alone (Fig. 5H; supplementary material Fig. S8J) (Peukert et al., 2011). Hence, we conclude that *lhx2* and *lhx9* function downstream of Fezf2 *in vivo*.

We next tested whether *lhx2* and *lhx9* act as an intermediary in the ability of Fezf2 to activate *ngn1* expression in the forebrain. Whereas *fezf2* morphant embryos were almost devoid of *ngn1* expression in the forebrain (Fig. 5I, arrowheads), *ngn1* expression was partially restored when *lhx2*/*lhx9* MOs were co-injected with *fezf2* MO (Fig. 5I; supplementary material Fig. S8K). We also found that, although *fezf2* overexpression in *chd*-neuralised explants induced the expression of the Wnt-responsive gene *xnr3* (Fig. 5J), this induction was attenuated by co-injection of *lhx2* and *lhx9* mRNAs (Fig. 5J). Moreover, the high level of *ngn1* induced by *fezf2* overexpression was also significantly attenuated by combined overexpression of *lhx2* and *lhx9* (Fig. 5K), suggesting that *lhx2* and *lhx9* are potent inhibitors of Wnt signalling in *Xenopus* neuroectoderm. Taken together, these findings suggest that *fezf2* inhibits the expression of the Wnt-repressive transcription factors *lhx2* and *lhx9*, thus promoting *ngn1* expression and, subsequently, neurogenesis in the forebrain.

## DISCUSSION

**Fig. 6.**
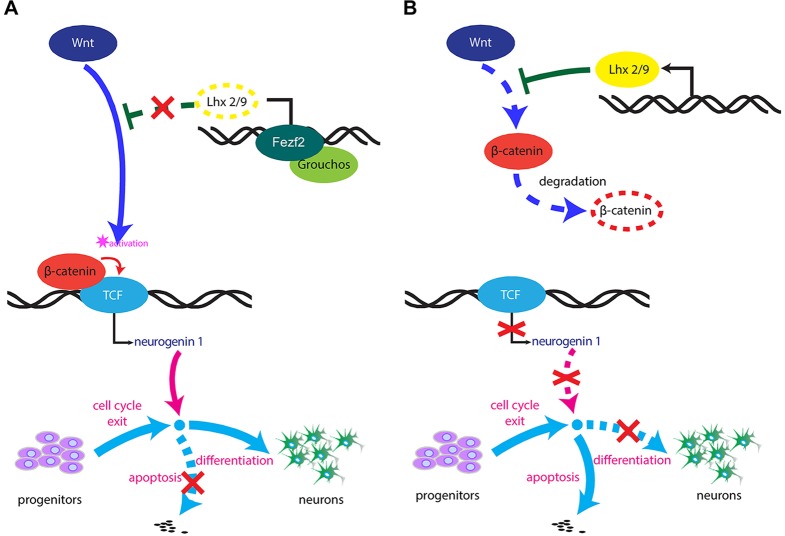
**Mechanistic model of Fezf2 function in the forebrain.** (A) In the presence of Fezf2. Fezf2 interacts with Groucho-family repressors and inhibits the expression of lhx2/lhx9. Consequently, β-catenin binds to the Tcf complex and Wnt signalling is activated, promoting the expression of neurogenin 1 and thus stimulating neuronal differentiation. (B) In the absence of Fezf2. Lhx2/Lhx9 inhibits Wnt signalling, resulting in the degradation of β-catenin. In the absence of β-catenin, the Tcf complex is maintained in a repressive state. This repressive Tcf complex inhibits neurogenin 1 expression, thus inhibiting neurogenesis. Progenitor cells that have exited the proliferation state cannot differentiate and thus enter apoptosis.

Growth and differentiation are crucial steps during the development and maturation of the forebrain. Here we propose that Fezf2 plays a crucial role during the regulation of forebrain neurogenesis through its ability to modulate Wnt/β-catenin signalling by a double-repressor model (Fig. 6A). Fezf2 in the forebrain area represses the expression of the Wnt-inhibitory genes *lhx2* and *lhx9*, thus permitting Wnt/β-catenin signalling to be activated. Consequent activation of Wnt/β-catenin signalling allows the Tcf complex to interact with β-catenin, freeing it from an inhibitory state (Lepourcelet and Shivdasani, 2002). As a result, *ngn1* expression is switched on (Hirabayashi et al., 2004; Israsena et al., 2004), thus allowing and promoting the differentiation of neural stem cells/progenitors into mature neurons (Hirabayashi et al., 2004; Jeong et al., 2006; Munji et al., 2011). By contrast, in the absence of Fezf2, Lhx2 and Lhx9 repress Wnt/β-catenin signalling (Peukert et al., 2011), which leads to impaired *ngn1* expression and increased apoptosis in committed neural stem cells/progenitors (Fig. 6B).

Stage-dependent regulation of Wnt/β-catenin signalling plays an essential role during anterior neural development. During the patterning stage, a low-to-high Wnt gradient across the anterior-posterior axis of the forebrain is required to establish telencephalon-diencephalon-midbrain identity (Heisenberg et al., 2001). By contrast, after the patterning stage is complete and neuronal differentiation begins, a number of Wnt ligands, including Wnt2b, Wnt5a/b, Wnt7b and Wnt8b, are expressed in ventral diencephalic and telencephalic areas (Quinlan et al., 2009). Expression of such Wnts activates Wnt/β-catenin signalling within the forebrain thereby promoting several events in neuronal differentiation, including the formation of cortical neurons, neural stem cells, basal progenitors and DA neurons (Castelo-Branco et al., 2003; Hirabayashi et al., 2004; Israsena et al., 2004; Kuwahara et al., 2010; Munji et al., 2011). In addition, an increase in Wnt activity in the mouse cerebrum has been reported to result in excessive neurogenesis, which further emphasises the promotional role of Wnt signalling in neurogenic activities (Seib et al., 2013). Our finding that Fezf2 may, at least in part, promote neurogenesis by its ability to activate Wnt signalling provides an additional layer to the exquisite temporal and spatial regulation of Wnt signalling that occurs during the differentiation phase of forebrain development.

Both positive and negative regulators are employed in modulating the transcriptional output of Wnt/β-catenin signalling in the forebrain, and a balance between agonising and antagonising regulatory mechanisms is employed to achieve this. Previous findings have identified several negative regulators of Wnt signalling, such as *barhl2* and *lhx2*/*lhx9*, in the forebrain (Hou et al., 2013; Juraver-Geslin et al., 2011; Peukert et al., 2011). However, no positive regulators have been identified to counterbalance the Wnt-inhibitory mechanisms in this area to ensure the proper temporal and spatial control of Wnt signalling and the consequent differentiation of progenitors after the initial patterning stage has been completed. Our finding that Fezf2 acts as a positive regulator of Wnt/β-catenin signalling through inhibition of *lhx2*/*lhx9* in the forebrain and, possibly, by repressing the expression of additional Wnt-inhibitory genes, provides insight into how balanced regulation of Wnt/β-catenin activity in the anterior forebrain occurs. Our results contradict a previous study that suggested that Fezf2 acts as a negative regulator of Wnt/β-catenin signalling during anterior neurogenesis (Jeong et al., 2007). However, the previous study did not assess the activity of Wnt signalling directly. Rather, it showed that misexpressing *fezf2* in the late gastrula stage zebrafish embryo results in the downregulation of *wnt1* expression. By contrast, our study investigated more directly the effect of *fezf2* upregulation and downregulation on Wnt/β-catenin activity using a number of assays, which all consistently showed that *fezf2* increased the activity of Wnt/β-catenin signalling. Furthermore, it is also notable that, when we assessed the effect of *fezf2* on the expression of genes encoding Wnt ligands, we found no effect in the cases of *wnt3a* and *wnt8b*, and an increase in the case of *wnt1*. Indeed, our results are consistent with established models that place Wnt/β-catenin signalling as an essential and stimulating factor that promotes the differentiation of neural stem cells/progenitors (Juraver-Geslin et al., 2011; Potok et al., 2008).

Functionally, several families of genes have been reported to be important for neuronal growth and differentiation in the forebrain, including the *iroquois* gene family (Gómez-Skarmeta and Modolell, 2002), *fezf1/2* (Hirata et al., 2006; Shimizu et al., 2010), *barhl2* (Juraver-Geslin et al., 2011) and *lhx2/lhx9* (Peukert et al., 2011). Whereas all previously identified genes act to inhibit neuronal growth and differentiation, *fezf2* plays a promotional role in these processes (Rouaux and Arlotta, 2010, 2013; Shimizu et al., 2010; Wang et al., 2011). Loss of *fezf2* results in various forebrain defects, including loss of monoaminergic neurons (Jeong et al., 2006; Levkowitz et al., 2003), disruption of diencephalon subdivisions (Levkowitz et al., 2003), and defects in reciprocal projections between thalamus and cerebral cortex (Komuta et al., 2007). It is noteworthy that all the above developmental defects can be attributed to insufficient or deficient neuronal differentiation, suggesting the pivotal role of *fezf2* in this process.

It is interesting that, although the expression of *fezf2* in the forebrain starts from the early patterning stage, its effect on Wnt/β-catenin signalling only becomes apparent from the tailbud stage at the onset of neuronal differentiation. One possibility that might account for this delayed function is that the Wnt-agonising activity of Fezf2 requires the participation of one or more unknown co-factors that are absent during the earlier neural patterning stage of development. It is also possible that, at the early stages, the anterior neuroectoderm is protected from Wnt signalling by several layers of Wnt-antagonising mechanisms.

A second interesting question is whether there are other transcription targets, in addition to *lhx2* and *lhx9*, that mediate some of the effects of Fezf2 during forebrain development. Since overexpression of *fezf2* can lead to Wnt activation in early stage embryos, when neither *lhx2* nor *lhx9* is yet expressed, it stands to reason that Fezf2 must be able to regulate the expression of additional targets that are responsible for the expanded activation of Wnt signalling in the early dorsoanteriorisation of embryos. Indeed, a recent study has revealed additional potential target genes of Fezf2 in cultured cortical progenitors (Lodato et al., 2014). Thus, an important future line of work will be to determine the function of these additional targets, including whether they also impinge on Wnt signalling.

Fezf2 has recently attracted great interest in the field of neural stem cell biology, as its expression marks multipotent progenitor cells and manipulating *fezf2* expression is able to provide a unique method for reprogramming postmitotic neurons within the mammalian neocortex (De la Rossa et al., 2013; Guo et al., 2013; Rouaux and Arlotta, 2010, 2013). In a series of unrelated studies, Wnt signalling, as the central signalling cascade regulated by *fezf2*, has been suggested to regulate neuronal differentiation and the assembly of neural connectivity and synapse formation and function (Munji et al., 2011; Oliva et al., 2013). Our studies, which link Fezf2 activity with Wnt signalling, suggest the tantalising possibility that the molecular mechanisms by which Fezf2 mediates lineage fate determination, reprogramming and plasticity might be mediated through its capacity to activate Wnt/β-catenin signalling. Our studies further suggest that modulating the activity of Wnt/β-catenin signalling, through the expression of *Fezf2*, might provide a powerful means of modulating the differentiation fates of neural stem cells, reprogramming postmitotic neurons or inducing neuronal plasticity.

## MATERIALS AND METHODS

### Sequences and constructs

Details of *X. tropicalis fezf2*, *tle4* and *aes* constructs, constructs for antimorphic studies and restriction enzyme-mediated integration (REMI) experiments using the pTransgenesis recombination system and associated cloning primers are provided in supplementary Materials and Methods and Table S2.

### mRNA microinjections

Microinjection of mRNA was performed as described previously, with *lacZ* mRNA co-injected as a tracer in some cases (Bourguignon et al., 1998). For further details see supplementary Materials and Methods.

### MO design and injection

MOs designed against *X. tropicalis* genes were supplied by Gene Tools. Typically, 10 ng MO was injected per *X. tropi*calis embryo at the 1- to 2-cell stage. Further details, including MO sequences, are provided in the supplementary Materials and Methods.

### Electroporation

Electroporation was performed as described (Falk et al., 2007). Briefly, 50 nl of 2 µg/µl plasmid mixtures were injected into the subventricular vesicles of stage 26 *Xenopus* embryos followed by electric pulses. Electroporated embryos were harvested at stage 30 for analysis.

### *In situ* hybridisation

Antisense digoxigenin-labelled RNA probes for whole-mount *in situ* hybridisation were prepared by T7 RNA polymerase-mediated transcription (Roche). X-Gal staining and *in situ* hybridisation were carried out as previously described (Bourguignon et al., 1998).

### Immunofluorescence, TUNEL staining and image processing

Fixed *Xenopus* embryos were cryosectioned for immunofluorescence (see supplementary Materials and Methods). Mouse c17.2 cells were grown in Lab-TEK II chambered slides (NUNC) and fixed with MEMFA. Details of c17.2 cell culture and transfection are provided in the supplementary Materials and Methods. Primary antibodies were: anti-Sox3 (a kind gift from the Klymokovsky lab; 1:1000) (Bonev et al. 2012), anti-MyT1 (1:1000) (Sabherwal et al., 2009) and mouse anti-acetylated tubulin (Sigma, T7451; 1:1000). Secondary antibodies were: anti-rabbit/mouse Alexa 488/568/647 (Invitrogen; 1:500). TMR Red (Roche) was used in TUNEL assays. Nuclei were stained with DAPI. Images were taken with a Nikon Eclipse 80i or an Olympus 2X81 confocal microscope and processed with ImageJ (NIH) software.

### *In vivo* luciferase assay

Briefly, 50 pg pTK-Renilla and 100 pg M50 TOPFlash (Addgene, 12456) or M51 FOPFlash (Addgene, 12457) (Veeman et al., 2003) were co-injected with 200 pg of either *fezf2* or control *lacZ* mRNA. Injected *Xenopus* embryos were collected at stage 10.5 and analysed with the DLR system (Promega). For further details see supplementary Materials and Methods.

### DEX induction of human glucocorticoid receptor fusion protein

The DEX-inducible VP16-Fezf2 construct was made by fusing the VP16-Fezf2 protein to the 3′-end of human glucocorticoid receptor (hGR) using *Xba*I-*Not*I restriction sites (Ryan et al., 2004). 500 pg of mRNA was injected into *X. laevis* embryos at the 1- to 2-cell stage. Animal cap explants (see supplementary Materials and Methods) were excised at stage 8 and allowed to develop until stage 12. A final concentration of 5 µg/ml CHX with or without 2 µM DEX in ethanol was used. Carrier alone (0.05% ethanol) was used as control. Animal cap explants were collected 2 h post treatment (Saka et al., 2000).

### Smad phosphorylation analysis

The phosphorylation status of signalling molecules in gastrula stage *X. laevis* embryos was determined by western blot analysis as described in the supplementary Materials and Methods.

### ChIP-qPCR

Chromatin co-immunoprecipitation (ChIP) was performed using a modification of published methods (Akkers et al., 2012; Blythe et al., 2009). Briefly, *X. tropicalis* embryos were injected with 50 pg FLAG-tagged *fezf2* mRNA, harvested at stage 15, crosslinked with 3.7% formaldehyde for 15 min and stored at −80°C until use. Approximately 300 embryos were used for each sample. Fezf2-binding fragments were enriched using anti-FLAG M2 antibody (Sigma) as described (Akkers et al., 2012). DNA regional enrichment was analysed by quantitative PCR (qPCR). qPCR primers are detailed in supplementary material Table S2. For further details see the supplementary Materials and Methods.

### Statistical analysis

For Sox3, MyT1 and TUNEL assays, positive cells were counted on two consecutive sections in the corresponding brain area for determination of the mean (Bonev et al., 2012); *n* is the number of individual embryos from at least three independent fertilisations and injections. For qPCR analyses, collected animal cap explants from individual experiments were pooled for RNA extraction, and all data were from at least three independent experiments (*n*=3), unless otherwise indicated. Statistical analysis was performed using GraphPad Prism software with either two-tailed unpaired Student's *t*-test (for two samples) or two-tailed unpaired one-way ANOVA (for multiple samples) and s.e.m. was calculated.

## Supplementary Material

Supplementary Material
