# Supplementary material for: Fezf2 promotes neuronal differentiation through localised activation of Wnt/β-catenin signalling during forebrain development
Source: Development. 2014 Dec 15;141(24):4794–805. doi: 10.1242/dev.115691 (PMC4299278; doi:10.1242/dev.115691)
Supplement: Supplementary Material [file supp_141_24_4794__index.html]

Supplementary Material 

# Fezf2 promotes neuronal differentiation through localised activation of Wnt/β-catenin signalling during forebrain development

## DEV115691 Supplementary Material

**Files in this Data Supplement:**

- Supplementary Material
