# Supplementary material for: Fezf2 promotes neuronal differentiation through localised activation of Wnt/β-catenin signalling during forebrain development
Source: Development. 2014 Dec 15;141(24):4794–805. doi: 10.1242/dev.115691 (PMC4299278; doi:10.1242/dev.115691)
Supplement: Supplementary Material [file supp_141.24.4794_DEV115691supp.pdf]

Sequences and constructs

The original *X. tropicalis fezf2* EST clone identified in the functional screen was TGas068O03. The coding sequence for *Xenopus tropicalis fezf2* was amplified by PCR and subcloned into the ClaI-SalI site of a modified pCS107 vector. *X. tropicalis tle4*-HA and a*es*-HA constructs were generously provided by Nancy Papalopulu (Roth et al., 2010). The list of primers used for cloning and qPCR analyses are provided in Supplementary Table S2. The qPCR primers used for identifying the temporal expression patterns of *fezf2* (*fezf2* and *rpl8*) were purchased from Applied Biosystems as TaqMan probes. HA and FLAG tags were added to the C-terminal end of Fezf2.

For the antimorphic studies, the VP16 transactivation domain was amplified from VP16-XBlimp (a kind gift by Christof Niehrs) and fused to the C-terminal zinc-finger domain of *fezf2* using HindIII to form VP16-Fezf2. Similarly, the Eve-repressor domain cloned from *Drosophila* cDNA was fused to the C-terminal zinc-finger domain of *fezf2* using HindIII to form Eve-Fezf2. Part of the En1 sequence (amino acid 30-34, FSIER) was removed with PCR and reconstructed to generate ΔEn1-Fezf2-pCS107.

The M50 TOPFlash (Addgene 12456) and M51 FOPFlash (Addgene 12457, contains mutated TCF/LEF binding sites and served as negative control) were originated from the Moon laboratory and purchased from Addgene. The pTK-Renilla plasmid (Promega) used as internal control.

Microinjections and embryo processing

Microinjection of mRNA was performed as described previously with lacZ mRNA co-injected as a tracer in some cases (Bourguignon et al., 1998). For microinjections, all pCS107-derived plasmids were linearised with Asc I (NEB); pCS2-derived

plasmids were linearised with Not I (NEB). pSPORT6-Msx1 was linearised with Not I. Ambion® mMessenger SP6 kit and MegaClear Spin columns were used for *in vitro* transcription of all mRNA transcripts.

Animal cap explants were isolated from mid-blastula (stage 8-8.5) embryos and cultured in ¾ NAM until specified stages. Collected embryos or explants were stored in Ambion® RNAlater solution and extracted using Qiagen RNEasy mini kit. For injection embryos or explants subjected to qPCR analysis, three replicates were used generate statistical results. qPCR analysis was performed using ABI high-capacity RNA-to-cDNA reverse transcription kit, ABI SYBR Fast or ABI TaqMan Fast reagent depending on experiment requirements. Data collection was performed on an ABI StepOnePlus machine.

RNA injection amounts for embryos and animal cap explants were as follows: *fezf2*: 100 pg; *lhx2*: 400 pg; *lhx9*: 400 pg; *ΔNTcf3*: 200 pg.

Morpholino (MO) design and injection

All MO experiments were performed in *X. tropicalis* with MOs specifically designed against the *X. tropicalis* genes and supplied by Gene Tools Inc. Some MOs were FITC-labelled to provide information on injection quality and to provide a measure of lineage labelling. The control MO was the standard Gene Tools control 25-mer (5'-CCTCTTACCTCAGTTACAATTTATA-3'). The *fezf2* e3i3 splicing MO sequence was 5'-AGGATTTCCCCCGTTCCGTACCTGT-3'. The *lhx2* i2e3 splicing MO sequence was ATCGCCTGTGCAATCAGAACCAGGA. The *lhx9* e1i1 splicing MO sequence was GGGTACACACGGCTACTTACTGTTT. For the primer sequences used in validating the MO function, refer to Supplementary Table 2. 10 ng of MO (5

ng x 2) was injected into each *X. tropicalis* embryo at the 1-2 cell stage unless specified.

Restriction enzyme mediated integration (REMI) and fluorescence stereoscope imaging

Construct assembly for REMI experiments was done using the pTransgenesis recombination system (Love et al., 2011). Briefly, either an NβT-tauGFP or an 7LEF-dEGFP (Denayer et al., 2006) cassette was placed in the p1 position such that both promoters transcribed in opposite directions. The 3kb *fezf2* core promoter was isolated and placed in p3 site. Several p3 constructs were made from a starting vector containing a 2A construct flanked by two pairs of restriction enzyme sites. The VP16-Fezf2 construct has been described. The stabilised β-catenin construct was made by removing the ~270 bp region encoding the N-terminal GSK3β binding domain (Aberle et al., 1997). The dominant-negative Tcf3 construct was made by removing the ~150 bp N-terminal β-catenin binding domain (Molenaar et al., 1996). The constitutively-active GSK3β was made by mutating the serine 9 into alanine thus preventing its inhibition by PI3K/AKT signalling (Stambolic and Woodgett, 1994; Sutherland et al., 1993). Only VP16-Fezf2 was inserted upstream of the 2A short peptide sequence, while the other inserts were all placed downstream of the 2A sequence. The p1, p2, and p3 constructs were integrated into the pDEST R4-R3 destination vector and subsequently linearised using Aat II (NEB) for integration. Standard REMI was performed as described (Ishibashi et al., 2008). After REMI, embryos were incubated in 16°C for 4 days (for *in situ* hybridisation) or 7 days (for immunofluorescence microscopy). Images were taken using a Leica M165FC stereoscope with colour CCD module attached.

Western blot and in vivo co-immunoprecipitation

For Western Blot analyses on the phosphorylation status of signalling molecules, each 7 injected *X. laevis* embryos were collected as a group at gastrula stage (10.5), extracted, and homogenized with SDS loading buffer for Western blot analysis. The Western blot protocol for analysing endogenous 1/5/8 and Smad2/3 has been described (Dorey and Hill, 2006). For RNA injection amounts, wild-type *fezf2*: 1ng; Eve-*fezf2*: 200pg; VP16-*fezf2*: 200pg.

For *in vivo* co-immunoprecipitation, 50 *X. laevis* embryos were injected with tagged mRNA at 1-2 cell stage as amounts followed: *fezf2-HA*, 500 pg; *ΔEh1-fezf2-HA*, 500 pg; *tle1-FLAG*, 500 pg; *tle2-FLAG*, 500 pg; *tle4-FLAG*, 500 pg; *tes-FLAG*, 500pg; *wt-Fezf2-FLAG*, 500 pg; Each injection was supplemented with GFP mRNA to achieve a total of 2 ng mRNA. Embryos were collected and homogenised at stage 10.5 using a modified IP-lysis buffer (50mM Tris-acetate pH 7.5, 300 mM NaCl, 1 mg/ml BSA, 2% NP40, protease inhibitor, and phosphatase inhibitor) followed by Freon extraction to remove excessive lipid in the extract. 10 µl of supernatant was kept as total input. 2 µg of anti-HA (Invitrogen) or anti-FLAG M2 (Sigma-Aldrich) antibody was added into 500µl extract and rotated for 2 hours at 4°C. Following the addition of 30 µl pre-incubated Protein A/G Sepharose (Santa Cruz), samples were rotated overnight at 4°C. Four rounds of washings were performed with washing buffer (50mM Tris-acetate pH 7.5, 300 mM NaCl, 1 mg/ml BSA, 0.5% NP40, protease inhibitor, and phosphatase inhibitor) and Sepharose beads were boiled with 20 µl of SDS-loading buffer for 10 minutes. The bead/buffer mix was loaded on a 10% SDS-PAGE gel.

Western blot was performed on a semi-dry system according to the manufacturer's instructions (Hoefer). PVDF membrane (Millipore) was blocked with 5% dry milk powder in TBS containing 0.1% Tween 20 for 1 hour followed by incubation with anti-Flag-HRP (1:10000, Sigma) or anti-HA-HRP (1:50000, Roche) antibodies in blocking buffer overnight. Following three washing steps in TBS containing 0.1% Tween 20. Blots were developed using Immobilon ECL reagents (Millipore).

Cell culture transfections

Mouse c17.2 neuronal progenitor cells (ATCC) were a kind gift from Nancy Papalopulu. Briefly, cells were maintained in DMEM supplemented with 10% FBS with penicillin/streptomycin. Mouse *Fezf2* and *ΔNTcf3* plasmids were cloned using primers described into pCS107 vector. Plasmid DNA constructs were transfected into cells plated in LAB-TEK II chambered slides (NUNC) and allowed to proliferate until 10-15% confluence before transfection using Lipofectamin 2000 following manufacturer's instruction (Invitrogen). After transfection, the cells were incubated in DMEM with 10% FBS for 1 day and medium was subsequently changed to DMEM with 1% FBS to allow differentiation for 4 days. The following amounts of plasmid were used: mouse *fezf2*, 1 µg; mouse *ΔNTcf3*, 1 µg. Each transfection was supplemented with empty pCS107 vector to a total of 2 µg DNA.

Cryosectioning

For *X. tropicalis* immunohistochemistry, embryos were fixed overnight at 4°C using MEMFA (3.7% formaldehyde, 1x MEM salts) and dehydrated in methanol. After rehydration using PBS, embryos were transferred into 15% fish gelatine/15% sucrose for 16 hours, sectioned on a cryostat (Leica CM3050) using 12 micron slice thickness and stained following standard protocols (Regad et al., 2007).

References

Aberle, H., Bauer, A., Stappert, J., Kispert, A. and Kemler, R. (1997). beta-catenin is a target for the ubiquitin-proteasome pathway. *EMBO J* **16**, 3797-3804.

Bourguignon, C., Li, J. and Papalopulu, N. (1998). XBF-1, a winged helix transcription factor with dual activity, has a role in positioning neurogenesis in Xenopus competent ectoderm. *Development* **125**, 4889-4900.

Denayer, T., Van Roy, F. and Vleminckx, K. (2006). In vivo tracing of canonical Wnt signaling in Xenopus tadpoles by means of an inducible transgenic reporter tool. *FEBS Lett* **580**, 393-398.

Dorey, K. and Hill, C. S. (2006). A novel Cripto-related protein reveals an essential role for EGF-CFCs in Nodal signalling in Xenopus embryos. *Dev Biol* **292**, 303-316.

Ishibashi, S., Kroll, K. L. and Amaya, E. (2008). A method for generating transgenic frog embryos. *Methods Mol Biol* **461**, 447-466.

Love, N. R., Thuret, R., Chen, Y., Ishibashi, S., Sabherwal, N., Paredes, R., Alves-Silva, J., Dorey, K., Noble, A. M., Guille, M. J., et al. (2011). pTransgenesis: a cross-species, modular transgenesis resource. *Development* **138**, 5451-5458.

Molenaar, M., van de Wetering, M., Oosterwegel, M., Peterson-Maduro, J., Godsave, S., Korinek, V., Roose, J., Destree, O. and Clevers, H. (1996). XTcf-3 transcription factor mediates beta-catenin-induced axis formation in Xenopus embryos. *Cell* **86**, 391-399.

Regad, T., Roth, M., Bredenkamp, N., Illing, N. and Papalopulu, N. (2007). The neural progenitor-specifying activity of FoxG1 is antagonistically regulated by CKI and FGF. *Nat Cell Biol* **9**, 531-540.

Roth, M., Bonev, B., Lindsay, J., Lea, R., Panagiotaki, N., Houart, C. and Papalopulu, N. (2010). FoxG1 and TLE2 act cooperatively to regulate ventral telencephalon formation. *Development* **137**, 1553-1562.

Stambolic, V. and Woodgett, J. R. (1994). Mitogen inactivation of glycogen synthase kinase-3 beta in intact cells via serine 9 phosphorylation. *Biochem J* **303 (Pt 3)**, 701-704.

Sutherland, C., Leighton, I. A. and Cohen, P. (1993). Inactivation of glycogen synthase kinase-3 beta by phosphorylation: new kinase connections in insulin and growth-factor signalling. *Biochem J* **296 (Pt 1)**, 15-19.

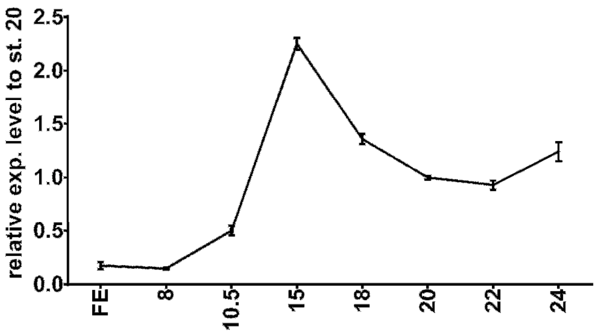

Figure S1. Temporal expression pattern of *X. tropicalis fezf2* revealed by qPCR analysis at different stages, including fertilised egg (FE), blastula (stage 8), gastrula (stage 10.5), neurula (stage 15-20), and tailbud (stage 22-24) (*n*=3 replicates). Error bars represent ±s. e. m.

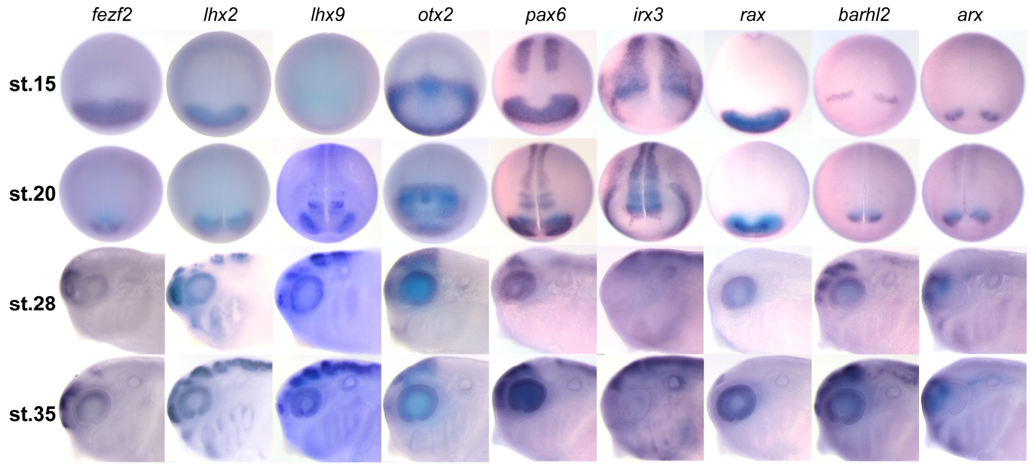

Figure S2. Comparison of expression pattern of *fezf2* expression relative to various nerual markers at different stages, related to Figure 1. Anterior is to the bottom in stage 15 and 20 embryos (frontal view), and to the left in stage 28 and 35 embryos (lateral view).

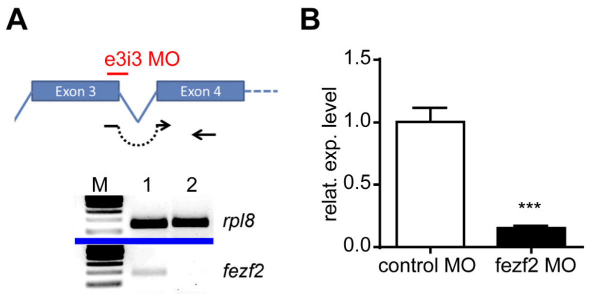

Figure S3. (A) Schematic of *fezf2* MO design and knockdown efficiency. The MO targets the exon3-intron3 junction (above) and semi-quantitative PCR analysis shows mature *fezf2* mRNA levels in stage 24 embryos (below), injected with control MO (1) or *fezf2* MO (2). Black arrow pair indicates the position of validation primers for RT-PCR analysis, the forward primer overlaps the exon3-exon4 junction. (B) qPCR results, *n*=3 replicates. In both cases, the *ribosomal protein L8* (*rpl8*) gene was used as an internal control.

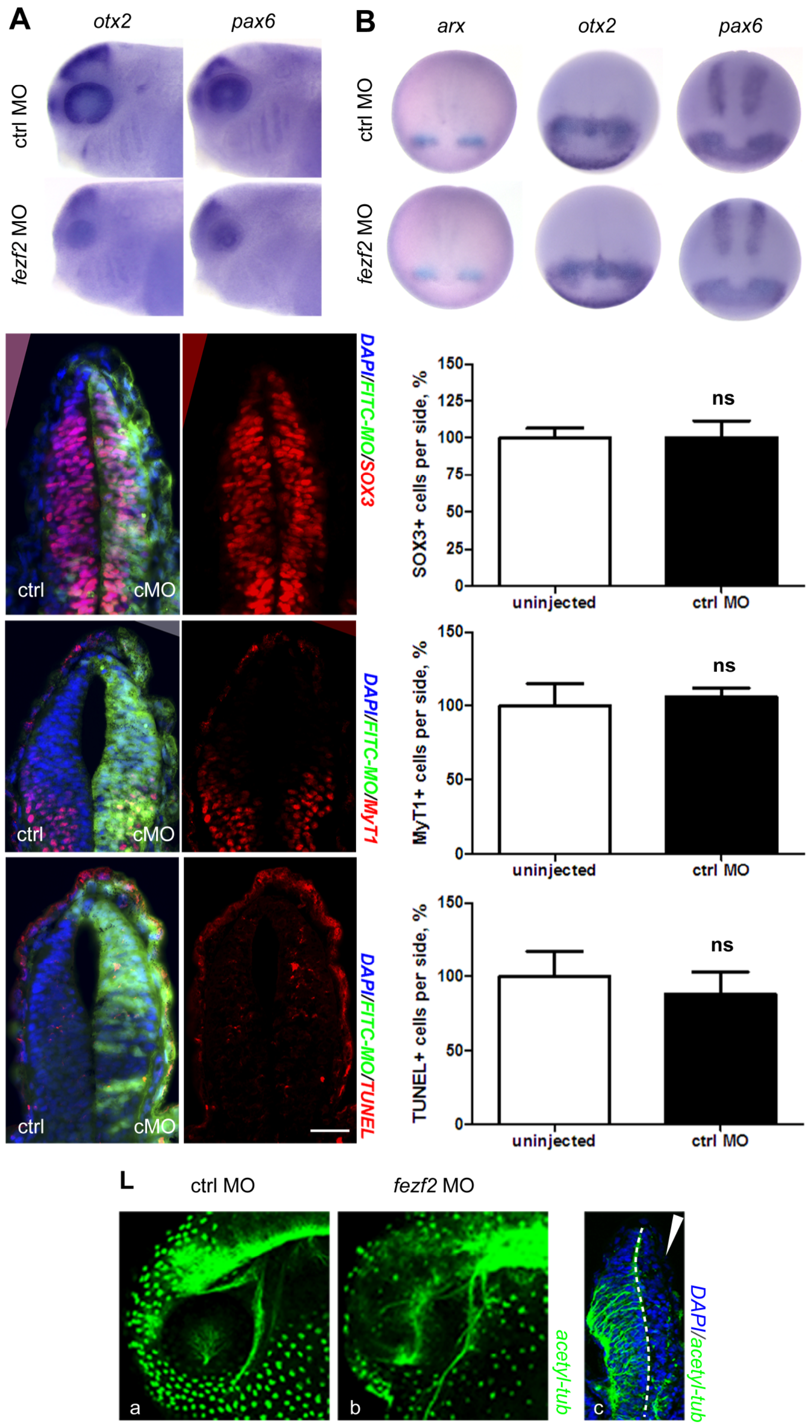

Figure S4 (related to Figure 1). *fezf2* knockdown, but not control MO knockdown, leads to defects in forebrain neuronal differentiation. (A) *fezf2* knockdown led to defects in forebrain development after initial patterning stage. Whole-mount *in situ*

hybridisation analyses on control versus *fezf2* MO injected stage 28 embryos, stained using the forebrain-specific markers, *otx2* and *pax6*. (B) *fezf2* knockdown did not cause defects in early forebrain patterning, as revealed by whole-mount *in situ* hybridisation on control versus *fezf2* MO injected stage 17 embryos, stained using the forebrain-specific markers *arx*, *otx2*, and *pax6*. Anterior is to the bottom. (C-H) 1 of 2 blastomeres at 2-cell stage was injected with control MO, cultured to stage 30, fixed sectioned transversely across the forebrain and stained via immunofluorescence for the transcription factor Sox3 (marker for neural progenitors) (C-D) or MyT1 (marker for differentiated primary neurons) (F-G). (C) Merged image. (D) Sox3 staining alone. (E). Statistical analysis of Sox3+ cells in control MO injected side relative to the uninjected side of the forebrain (n=3 embryos, no significant difference). The uninjected side has been normalised to 100%. (F) Merged image. (G) MyT1 staining alone. (H) Statistical analysis of MyT1+ cells in control MO injected side relative to the uninjected side of the forebrain (n=3 embryos, no significant difference). The uninjected side has been normalised to 100%. (I-K) Transverse sections were prepared as for panels C-D and then processed for TUNEL staining. (I) Merged image. (J) TUNEL-positive cells alone. (K) Percentage of the TUNEL-positive cells in the control MO injected side compared to the uninjected side (n=3 embryos). In all cases, the FITC tag on the control MO was used to identify the injected side; DAPI was used to stain nuclei. Error bars represent ±s. e. m. Scale bar: 25 μM. ns: not significant. (L) (a-b) Immunofluorescence staining using the neuronal differentiation marker, N-tubulin, on stage 32 embryos injected with 10 ng of control or *fezf2* MO. (a) control MO; (b) *fezf2* MO, showing a strong reduction of N-tubulin staining in the rostral area. (c) Sections of stage 32 embryos at the prethalamus level and stained with N-tubulin antibody. Embryos were injected with 5 ng of *fezf2* MO into one of the two

blastomeres at the 2-cell stage. A strong reduction of N-tubulin staining was observed in the injected side. Arrowhead: injected side. DAPI was used to provide global visualisation on the head structure. In all cases, error bars represent ±s. e. m.

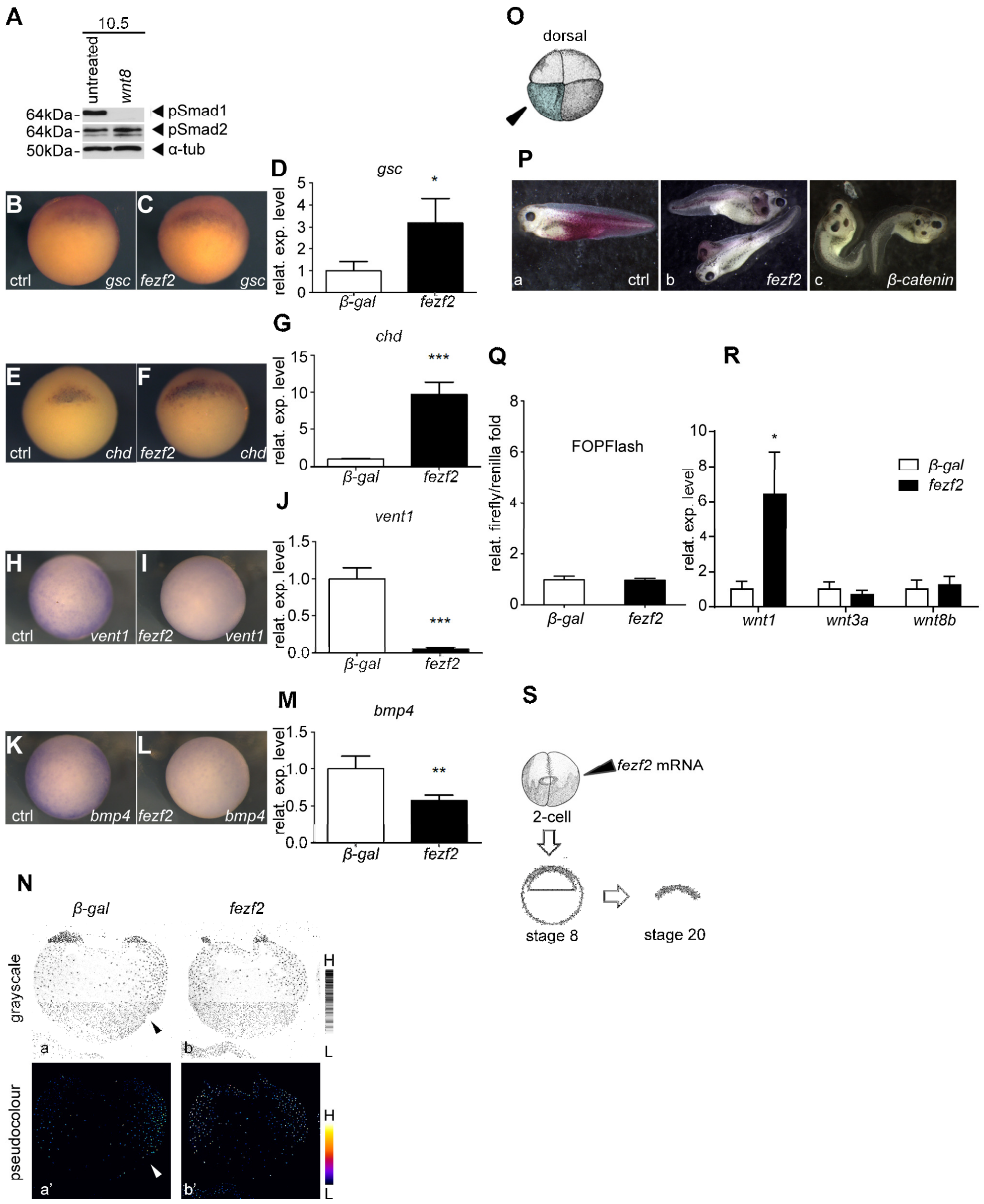

Figure S5 (related to Figure 2). *fezf2* promotes Wnt/β-catenin signalling. (A) *wnt8* mis-expression inhibited BMP signalling while activating TGF-β/Nodal signalling. 50 pg of either nuclear β-gal mRNA or *wnt8a* mRNA was injected into *X. laevis* embryos

at 1-2 cell stage and collected at the gastrula stage (10.5) for Western blot analysis. Phospho-Smad 2/3 antibody (TGF-β/Nodal signalling) or phospho-Smad 1/5/8 (BMP signalling) were used assay for the activation states of TGF-β/Nodal or BMP signalling. A significant increase on the phosphorylation level of Smad 2/3 was observed together with significant decrease on the phosphorylation level of Smad 1/5/8 was seen in *wnt8* mRNA injected embryos. (B-G) *fezf2* mis-expression promotes Wnt-responsive dorsal markers *goosecoid* and *chordin* expression. In all cases *ribosomal protein L8* (*rpl8*) gene was used as internal control for qPCR analysis. (B-D) *goosecoid* expression. (B) Uninjected control embryo. (C) *fezf2* mRNA overexpressing embryo. (D) qPCR analysis, showing an increase of *goosecoid* expression (n=3 replicates, P<0.05). (E-G) *chordin* expression. (E) Uninjected control embryo. (F) *fezf2* mRNA overexpressing embryo. (G) qPCR analysis, showing an increase of *chordin* expression (n=3 replicates, P<0.001). (H-M) *fezf2* mis-expression inhibits ventral markers *vent1* and *bmp4* expression. Embryo treatments are same as in (B-G). (H-J) *vent1* expression. (H) Uninjected control embryo. (I) *fezf2* mRNA overexpressing embryo. (J) qPCR analysis, showing a decrease of *vent1* expression (n=3 replicates, P<0.001). (K-M) *bmp4* expression. (K) Uninjected control embryo. (L) *fezf2* mRNA overexpressing embryo. (M) qPCR analysis, showing a decrease in *vent1* expression (n=3 replicates, P<0.01). (N) *fezf2* mis-expression leads to nuclear accumulation of β-catenin. 250 pg of either nuclear β-gal (control) or *fezf2* mRNA was injected at 1-2 cell stage and section made at stage 10, DAPI mask used to reveal nuclear content and the localisation of nuclear β-catenin after masking, showing increased nuclear enrichment of β-catenin in *fezf2* mis-expressed embryos. Both grayscale and pseudo-colour images have been used to show the level of β-catenin presence in the nucleus. (a and a’) control; (b and b’) *fezf2* injected embryos.

Arrowhead: blastopore lip. (O-P) Axis duplication assay. (O) Schematic representation of ventral blastomere injection. 250 pg of mRNA, together with nuclear β-gal mRNA as tracer, was injected into one of the ventral blastomere (arrowhead) at the 4-cell stage. Embryos were fixed at approximately stage 38 and stained with Red-gal. D: dorsal side. (P) Results of axis duplication assay. (a) Control embryo, nuclear β-gal mRNA injection only. (b) 250 pg *fezf2* mRNA. (c) 250 pg β-catenin mRNA. (Q) Dual-luciferase assay on control FOPFlash plasmid containing mutated TCF-binding sites. pTK-renilla was used as endogenous control. (R) qPCR analysis on *wnt1*, *wnt3a*, and *wnt8b* in nuclear β-gal (control) or *fezf2*-injected neuralised animal cap explants aged to stage 15. n=3 replicates. * $P<0.05$. In all cases, error bars represent ±s. e. m. (K) Schematic representation of animal cap explant assay. mRNA at different combinations were injected at the 1-2 cell stage and allowed to develop until the mid-blastula stage (stage 8). Animal cap explants were excised at stage 8 and allowed to develop to stage 20, at which point they were collected for qPCR analysis.

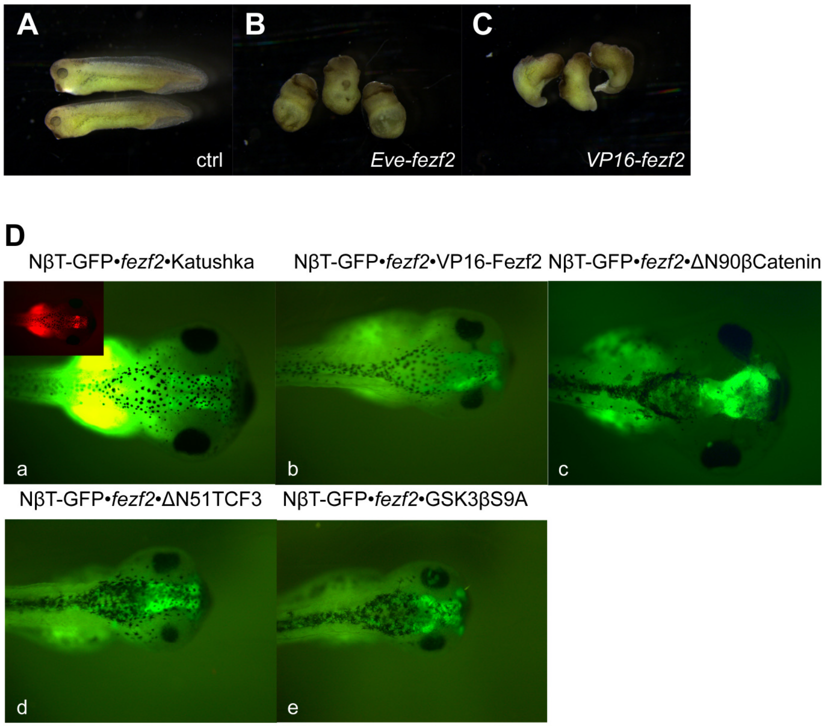

Figure S6 (related to Figure 3). *fezf2*-regulated endogenous level of Wnt/β-catenin signalling governs forebrain neurogenesis in transgenic embryos. (A-C) Phenotypic effect of antimorphic Fezf2. (A) nuclear β-gal (control) mRNA, (B) *fezf2* mRNA, showing strongly dorsal-anteriorized (DAI 8-9) embryos. (C) VP16-*fezf2* mRNA, showing strongly ventralised (DAI 1-2) embryos. (D) GFP expression in the forebrain of embryos from different transgenic lines at day 7, top view. (a) NβT-GFP·*fezf2*·Katushka. Green: GFP; red: Katushka (inset). (b) NβT-GFP·*fezf2*·VP16-Fezf2. (c) NβT-GFP·*fezf2*·ΔN90βcatenin. (d) NβT-GFP·*fezf2*·ΔN51Tcf3. (e) NβT-GFP·*fezf2*·GSK3βS9A.

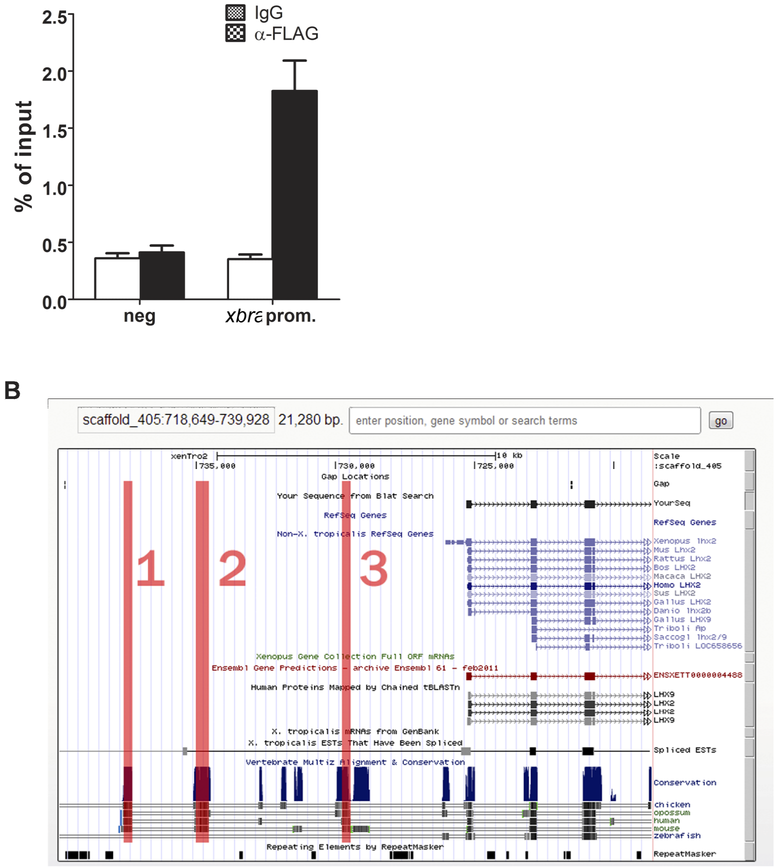

Figure S7 (related to Figure 5). Demonstration of ChIP-qPCR efficiency and Fezf2 binding site. (A) ChIP-qPCR result of FLAG-tagged FoxH1 using IgG (control) or anti-FLAG antibody, showing enrichment in the proximity of *branchyury (xbra)* promoter. (B) Schematics of the three highly conserved regions screened by ChIP-qPCR, highlighted areas have been screened using primers designed against each of the different regions.

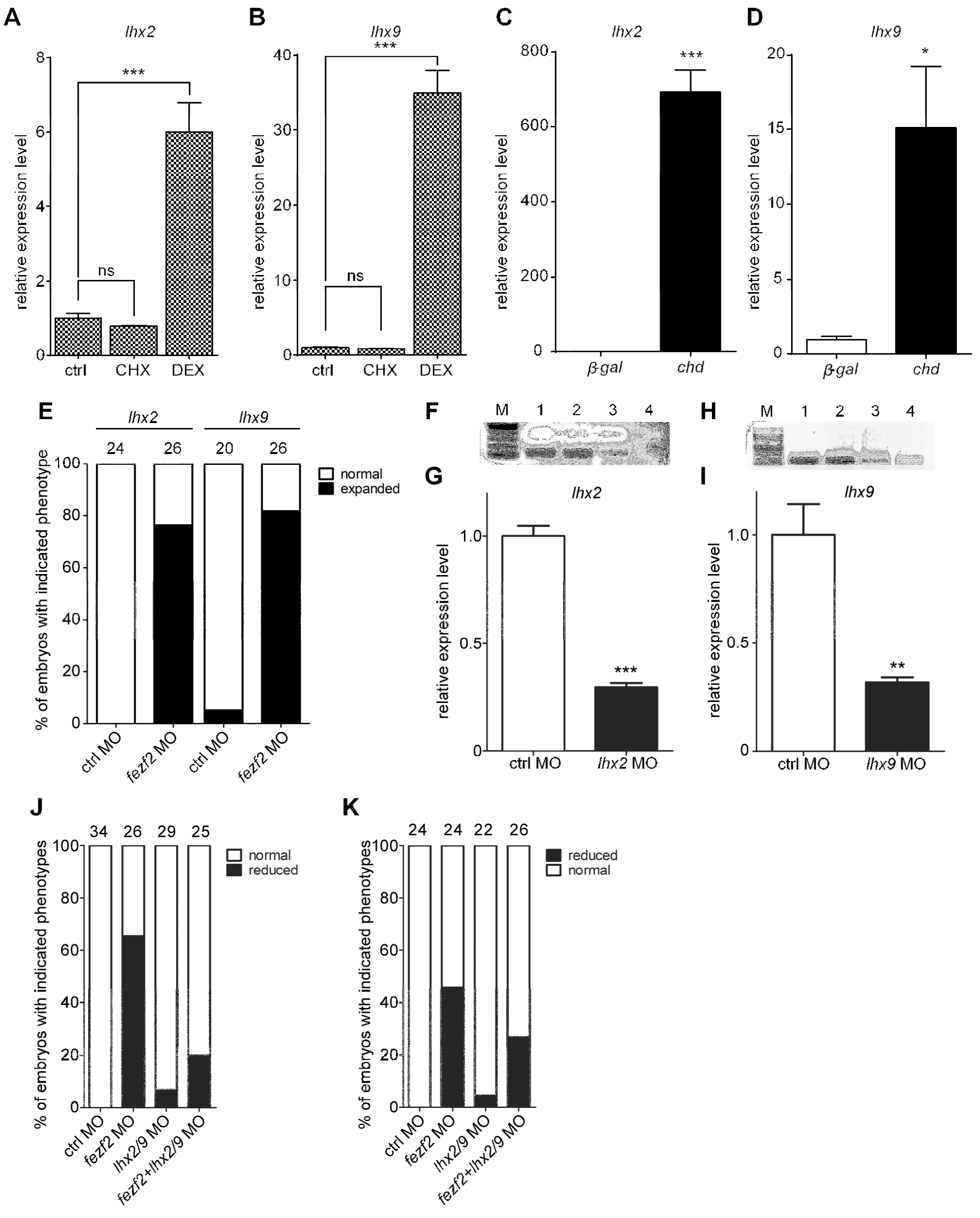

Figure S8 (related to Figure 6). Fezf2 represses the activities of *lhx2* and *lhx9*. (A-B) Control experiments showing the expression of *lhx2* and *lhx9* in stage 12 animal cap

explants injected with p3hGR-VP16-Fezf2, either left untreated, or treated with either CHX or DEX alone. qPCR results of (A) *lhx2* and (B) *lhx9* are shown (*n*=3 replicates, *P*<0.001). *rpl8* was used as an internal control. (C-D) Stage 20 animal cap explants neuralised by *chordin* mRNA expressed high level of *lhx2* and *lhx9*. qPCR results of (C) *lhx2* and (D) *lhx9* are shown (*n*=3 replicates, *P*<0.001 and *P*<0.05). *rpl8* was used as an internal control. (E) Bar graph of the quantification and statistics of phenotypes from control or *fezf2* morphant groups regarding the expression of *lhx2* and *lhx9*. (F-G) Validation of *lhx2* i2e3 MO. (F) Validation by RT-PCR. M: marker. Lane 1-2: *rpl8*; 1: control MO; 2: *lhx2* MO. Lane 3-4: *lhx2*; Lane 3: control MO, Lane 4: *lhx2* MO. (G) Validation by qPCR. *rpl8* was used as an internal control in both cases. *n*=3 replicates, *P*<0.001. (H-I) Validation of *lhx9* e1i1 MO. (H) Validation by RT-PCR. M: marker. Lane 1-2: *rpl8*; 1: control MO; 2: *lhx9* MO. Lane 3-4: *lhx9*; Lane 3: control MO, Lane 4: *lhx9* MO. (I) Validation by qPCR. *rpl8* was used as an internal control in both cases. *n*=3 replicates, *P*<0.01. (J) Bar graph of the quantification and statistics of phenotypes from different morphant groups regarding *arx* expression. (K) Bar graph of the quantification and statistics of phenotypes from different morphant groups regarding *ngn1* expression. In all bar graphs, numbers above each bar represent counts of embryos examined in each group. Error bars represent ±s. e. m.

Table S1. Results of the axis branching assay.

Click here to Download Table S1

Table S2. Primers used in making constructs and qPCR analysis.

Click here to Download Table S2
